# Reply to Ismayilli et al. Comment on “Megat Ramli et al. A Systematic Review: The Role of Artificial Intelligence in Lung Cancer Screening in Detecting Lung Nodules on Chest X-Rays. *Diagnostics* 2025, *15*, 246”

**DOI:** 10.3390/diagnostics16020209

**Published:** 2026-01-09

**Authors:** Puteri Norliza Megat Ramli, Azimatun Noor Aizuddin

**Affiliations:** 1 Institut Kanser Negara, Ministry of Health, Putrajaya 62250, Malaysia; p131318@siswa.ukm.edu.my; 2 Department of Public Health Medicine, Faculty of Medicine, Universiti Kebangsaan Malaysia, Cheras 56000, Wilayah Persekutuan Kuala Lumpur, Malaysia

We would like to sincerely thank you for your thoughtful and very helpful comments on our systematic review titled “A Systematic Review: The Role of Artificial Intelligence in Lung Cancer Screening in Detecting Lung Nodules on Chest X-Rays” [1]. Your remarks point out an important issue in the use of AI for screening, which is that the performance of an algorithm cannot be viewed alone. It must also be considered together with what happens afterwards in the clinical pathway, especially when false-positive cases increase. This point was also mentioned in the recent work by Geppert et al. 2024, who noted that even a small rise in false-positive rate may cause quite heavy burden in settings where disease prevalence is low [2].

In our review, we focused mainly on diagnostic accuracy findings from 34 studies, which were quite different from each other. Because of this, we found a wide range of values for specificity and positive predictive value (PPV). Some studies, especially those in real-world screening cohorts, showed PPV as low as 1.3% [3], while others using more controlled datasets reported PPV above 70% [4]. As you highlighted, this kind of variation is not a small matter in low-prevalence environments. Even a modest increase in false positives can mean more CT scans, additional clinic appointments, and also more anxiety for patients. This pattern is in line with the findings by Maiter and Hocking [5], who only observed PPV of 5.5% in their screening cohort, suggesting the possibility of over-investigation.

Although our review did mention these issues in a more indirect way—such as discussing threshold optimisation and the role of AI as a supplementary tool—we admit that the broader system implications of false positives should be discussed more clearly. Like you pointed out, any improvement in sensitivity must be balanced with how resources are used in real practice, how the workflow is affected, and also how patients actually feel the impact.

We also appreciate your comment regarding study designs such as stepped-wedge cluster randomised trials. These approaches offer something beyond the usual diagnostic accuracy metrics because they can capture real-world workflow changes, imaging utilisation, and patient outcomes after AI is introduced. Such pragmatic designs are needed if we want to connect technical performance with actual clinical value.

Your practical recommendations for implementing AI in screening pathways are also important, and they match well with what we summarised in the manuscript:

1. Using AI as a second reader, especially in difficult anatomical areas where nodules are commonly missed, is consistent with the sensitivity improvements seen particularly among less experienced readers.
2. AI performance needs to be assessed across different clinical settings, since inconsistency between environments can disturb the balance between sensitivity and false positives.
3. Continuous monitoring and user-focused system design are necessary so that the AI tool can function properly in various practice situations.

We agree that bringing in these governance elements will help provide a more complete understanding of AI’s role in lung cancer screening, not only from the technical aspect but also its clinical value.

Once again, we truly appreciate your thoughtful and constructive feedback. Your insights help to strengthen the field of AI research and also the safe and meaningful adoption of AI systems in clinical services. We believe that combining diagnostic accuracy evidence with real-world implementation research, as you suggested, will further support the development of AI-assisted lung cancer screening.
